# What comes before the cyst-early pulmonary Langerhans cell histiocytosis manifestation

**DOI:** 10.36416/1806-3756/e20250493

**Published:** 2026-05-22

**Authors:** Felipe Marques da Costa, Milena Tenório Cerezoli, Augusto Kreling Medeiros

**Affiliations:** 1. Serviço de Pneumologia, Hospital Beneficência Portuguesa de São Paulo, São Paulo (SP), Brasil.; 2. BP Medicina Diagnóstica, Hospital Beneficência Portuguesa de São Paulo, São Paulo (SP), Brasil.

**Figure 1:**
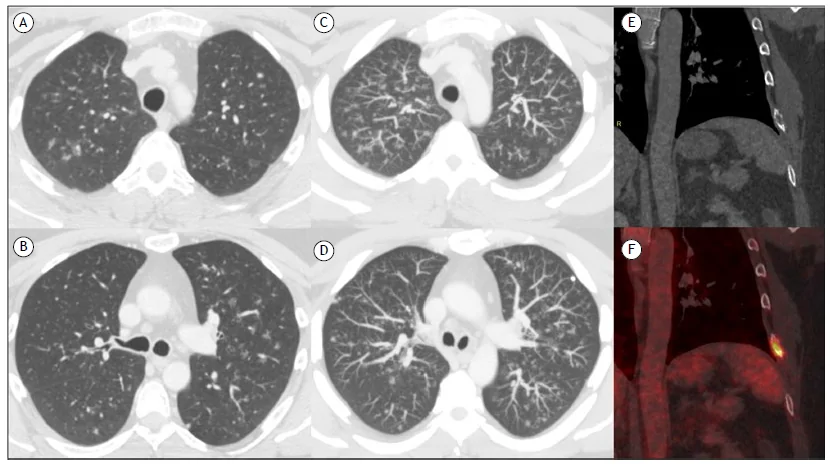
In A and B, axial chest CT images at the upper and middle lung zones demonstrate multiple relatively well-circumscribed nodular opacities, predominantly distributed along the peribronchovascular interstitium, with variable attenuation ranging from ground-glass to mildly solid components. In C and D, axial maximum-intensity-projection (MIP) reconstructions highlight the extent and peribronchovascular predominance of these nodular opacities. In E and F, CT and fused PET/CT images show an aggressive osteolytic lesion of the left eighth rib, with cortical breakthrough and marked hypermetabolic activity (SUVmax = 8.7). In G and H, CT and fused PET/CT images reveal a lytic lesion in the right intertrochanteric femur characterized by internal septations, foci of mineralized matrix, and a peripheral sclerotic rim, demonstrating moderate radiotracer uptake (SUVmax = 5.8) without cortical destruction.

A 41-year-old male smoker presented to the emergency department with severe localized chest pain over the left eighth rib. The results of the physical examination were unremarkable. Chest CT revealed bilateral centrilobular pulmonary nodules predominantly distributed in the upper zone and a lytic lesion involving the left eighth rib (Figure 1). 18F-FDG PET/CT revealed increased glycolytic activity within the rib lesion. Surgical lung and rib biopsies revealed morphological and immunohistochemical features consistent with pulmonary Langerhans cell histiocytosis (LCH). 

Pulmonary LCH is a rare condition with an adult prevalence of approximately 1-2 per million population and is closely associated with cigarette smoking. It can occur independently or as part of multisystem LCH, which may involve extrapulmonary sites, such as the bones.^1^ Chest CT typically reveals small centrilobular nodules in the early stages, which may cavitate and evolve into irregular cysts, primarily in the upper and mid-lung regions.^2^ ^,^ ^3^ For a definitive diagnosis, tissue samples showing Langerhans cells are essential, open lung biopsy being the gold standard, whereas bronchoscopy yields limited results. The primary management strategy is smoking cessation, with systemic therapy reserved for progressive disease cases.^3^
